# Induction of labor with oxytocin in pregnancy with low-risk heart disease: A randomized controlled trial

**DOI:** 10.4274/tjod.galenos.2019.59932

**Published:** 2020-02-28

**Authors:** Yogita Dogra, Vanita Suri, Neelam Aggarwal, Ravi Kant Dogra

**Affiliations:** 1 All India Institute of Medical Sciences, Clinic of Obstetrics and Gynecology, New Delhi, India; 2 Postgraduate Institute of Medical Training and Research, Clinic of Obstetrics and Gynecology, Chandigarh, India; 3 Indira Gandhi Medical College, Department of Anesthesiology, Shimla, India

**Keywords:** Heart disease, labor, induction, caesarean, delivery

## Abstract

**Objective:**

To compare maternal and perinatal outcomes in pregnant women with underlying heart disease who underwent induction of labor with those who had spontaneous labor.

**Materials and Methods:**

A total of 50 pregnant women with heart disease who were registered in cardio-obstetric clinic were recruited consecutively between 38-41 weeks’ gestation. Patients with favorable Bishop scores at 38 weeks were randomized into two groups. Induction of labor with oxytocin was performed in one group, and the second group underwent spontaneous onset of labor. Descriptive analysis in terms of mean, standard deviation, and percentage was performed. Unpaired t-test was applied for comparison of two groups using SPPS statistical software.

**Results:**

No significant difference in the rate of maternal complications was observed between the two groups. No cardiac complications were reported in pregnant females who underwent induction of labor. Fifty-two percent of patients delivered during workday hours when labor was induced, whereas only 24% of pregnant women delivered during working hours who underwent spontaneous delivery. No maternal or neonatal deaths were reported.

**Conclusion:**

Induction of labor with oxytocin is a relatively safe procedure in women with heart disease, it does not result in any cardiac complications. More patients delivered during daytime when electively induced, which minimized the maternal and fetal risks because obstetric, anesthesiologist, cardiologist, and perinatologist specialists are readily available during the daytime.

**PRECIS:** Elective induction of labor with oxytocin is a relatively safe procedure in women with low-risk heart disease.

## Introduction

About 1% of all pregnancies are complicated by cardiovascular disease, and the incidence is perpetually rising with the advancement of the medical field, as raised treatment standards allow more patients with heart disease to reach childbearing age^(1,2)^. As is evident, during pregnancy, labor, delivery, and the postpartum period, hemodynamic alterations are bound to occur^(3)^, the odds of maternal mortality and morbidity surge considerably with a compromised heart^(4)^, heart disease, which complicates pregnancy, may be either congenital or acquired. Cyanotic heart diseases, diminished systemic ventricular function, complex congenital heart disease, left ventricular outflow tract obstruction, pulmonary hypertension, or mechanical valves are at maximum risk of developing complications during pregnancy^(5)^. Maternal mortality varies directly with the functional class of heart disease: 0.4% for New York Heart Association (NYHA) classes I and II, and 6.8% for classes III and IV. The maternal functional class also influence the fetal mortality, varying from zero for class I to 30% for class IV^(6,7)^. The management of delivery in such cases is vital to the survival of both mother and baby and should be performed under the presence of a team of obstetricians, cardiologists, anesthetists, and perinatologists. Several studies have authenticated that cesarean section is accomplished more often in women with heart disease than in the healthy population^(8,9)^. However, the cesarean section is associated with more blood loss and higher thromboembolic and infection risks; therefore, vaginal delivery is favored in such cases, and cesarean section is reserved for obstetric indications^(10)^. Vaginal delivery with low-dose regional analgesia and careful fluid management is the preferred mode of delivery in most cases^(11)^.

Vaginal deliveries can either be spontaneous or induced. An upsurge has been observed in the rate of labor induction among women with term pregnancies during the past 10-15 years^(12,13)^. However, labor must be induced with caution in women with heart disease because the physiologic rise in cardiac output may burden an already compromised heart^(14)^. It has been observed that majority of the hospital deliveries occurring during the night hours are associated with adverse perinatal outcomes^(15)^. The rates of emergency caesarean sections and neonatal intensive care unit (NICU) admissions are also increased considerably in the night-shift period^(16)^. Labor induction for pregnant women with underlying heart disease is often offered to control the timing and setting of labor, to ensure the presence of a team of cardiologists, obstetricians, anesthetists, and perinatologists^(17,18)^.

There is limited literature comparing induction of labor and spontaneous labor in such a group of patients. So far, the induction of labor has been performed for obstetric indications only, and not the heart disease per se. The aim of this prospective randomized controlled study was to compare maternal and perinatal outcomes in these two groups, which might help in formulating guidelines regarding the management of these patients and controlling the timing of delivery in these patients, to avoid night deliveries.

## Materials and Methods

The present prospective randomized controlled trial was conducted in the Department of Obstetrics and Gynecology at a tertiary care referral and research institute of Northern India. Clearance from the institute’s Ethical Committee was obtained for the study and was performed following ethical standards. A total of 50 pregnant women with heart disease who were registered in the cardio-obstetric clinic were recruited consecutively between 38-41 weeks’ gestation after obtaining informed consent. Randomization was performed using a computer-generated random number tables in a 1:1 ratio and sealed opaque envelopes. Women with a known case of heart disease with NYHA class I-II and cephalic singleton gestation were included in the study. Patients with previous cesarean section, primary pulmonary hypertension, Eisenmenger syndrome, Marfan syndrome, left heart obstruction, prior cardiac event or arrhythmia, malformed fetus, severe anemia (<7 g/dL), intrauterine fetal death, other obstetric indications for induction of labor, and patients on anticoagulation were excluded from the study. The cervical assessment was performed using Bishop^(19)^ scores at 38 weeks. A sealed envelope was opened if a score of more than or equal to 6 was found and patients were allocated to the assigned group and admitted electively. Patients with a score of less than six were reassessed after one week till 41 weeks, and randomization was performed when the Bishop score was more than or equal to 6. The two groups were as follows:

Group I: Induction of labor was started in the morning with oxytocin. An infusion of 30 U oxytocin diluted in 500 mL normal saline was prepared and given through an infusion pump at an initial rate of 3 mU/min. Subsequently, the dose was increased by 3 mU/min every 45 min till adequate uterine contractions were established (at least 3-5 uterine contractions every 10 minutes).

Group II: Patients waited for spontaneous onset of labor.

In both groups, progress of labor was monitored similarly. Multidisciplinary care from cardiologists, obstetricians, and anesthetists was provided to all patients admitted to the labor room. Epidural analgesia was provided wherever feasible, or an injection of morphine 2-5 mg was given intravenously. Epidural analgesia was provided using 0.0625% bupivacaine with a 25 microgram fentanyl bolus of 6-8 mL, followed by infusion of 0.1% bupivacaine with two microgram/mL fentanyl at 6-8 mL/hr. Infective endocarditis prophylaxis was given before epidural analgesia, at spontaneous rupture of membranes or when the delivery was imminent. The second stage of labor was cut short wherever needed. The third stage of labor was actively managed. Patients were observed for postpartum hemorrhage and infections in the immediate postpartum period.

The outcome measures were: obstetric outcome in terms of duration of labor, mode of delivery, and rate of cesarean section with indication; maternal outcomes in terms of postpartum hemorrhage, infection, development of cardiac complications such as pulmonary edema, heart failure or pulmonary embolism, and maternal death; and neonatal outcomes in terms of apgar score, need for admission to the NICU, and neonatal death. Descriptive analysis in terms of mean, standard deviation and percentage was performed. The statistical analysis was conducted using the chi-square and Fisher’s exact test for categorical data. The unpaired t-test was used for the comparison of two groups because the data were normally distributed. All statistical tests were two-sided and performed at a significance level of α=0.05.

## Results

The study group included 50 pregnant women with documented cardiac disease, of whom 25 underwent induction of labor (group I) and 25 had the spontaneous onset of labor (group II). The distribution of pregnant women in terms of gravida, gestational age of delivery, and type of preexisting cardiac disease is depicted in Table 1. The majority had a gestational age of 38-39 weeks at delivery, with mean gestational age 38.5±0.735 weeks in group I and 38.5±0.657 weeks in group II. Ninety-six percent of patients in each group were of NYHA class I type of cardiac disease with a preponderance of rheumatic heart disease (64% in group I and 68% in group II) as compared with congenital heart disease.

The obstetric outcomes in terms of duration of labor, the timing of delivery, mode of delivery, and rate of cesarean sections with indications is represented in Table II. The mean duration of labor in group I was 7.56±4.806 hours, whereas in group II, it was 7.35±7.440 hours (p=0.906). There was no significant difference observed between the groups in the cesarean delivery rate. The incidence of cesarean section in cardiac pregnancies was 6% in this study. The indications included fetal bradycardia (n=1), meconium-stained liquor (n=1), and deep transverse arrest (n=1). However, none had a cesarean section for a cardiac indication. Nine women (18%) had an instrumental vaginal delivery, either by forceps (16%) or ventouse (2%).

There was no significant difference in the rate of maternal complications observed between the two groups. None of the patients had postpartum complications, and there were no cardiac complications reported such as pulmonary edema or congestive heart failure. There were no maternal deaths in this study.

The neonatal outcomes in both groups are depicted in Table III. The majority of babies in this study had a birth weight between 2.6-3.0 kg (52%). There was no significant difference in the birth weights of the newborns between the two groups. There were no stillbirths, and none of the babies had an Apgar score <7 at 5 minutes.

## Discussion

Minimizing maternal and fetal risks in a pregnant woman with associated heart disease requires the mutual efforts of experienced specialists who are familiar with their management. This team should involve obstetricians, cardiologists, anesthetists, and perinatologists. The timing and mode of delivery, anesthesia and analgesia, cardiac monitoring, and place of delivery should be planned well beforehand. So far, the induction of labor has been performed for obstetric indications only and not the heart disease per se. There is limited literature comparing the outcomes of inductions of labor and spontaneous labor in pregnant women with cardiac disease.

This prospective randomized controlled study was designed to compare maternal and perinatal outcomes between electively induced labor and spontaneous labor in patients with heart disease. This study aimed to determine whether any statistical difference existed between the rate of cesarean delivery, and maternal and neonatal complications in women who did and did not undergo induction of labor. The hypothesis was that the induction of labor was a safe procedure in women with cardiac disease if no significant difference could be demonstrated.

In the present study, 50 consecutive women with heart disease were recruited between 38-41 weeks’ gestation after ascertaining eligibility criteria. The decision to induce labor was made jointly by the cardiologist and the obstetric team based on the clinical assessment of each patient’s cardiac status and obstetric indications. Induction was performed using oxytocin according to our departmental protocol.

On analyzing the results, it was observed that the majority (34/50) of women were multigravidas. We observed that more patients had rheumatic (66%) rather than congenital heart disease (24%). This is in contrast to recent reports of the west, where congenital heart disease was reported as the primary cause of heart disease in pregnancy. Oron et al.^(20)^ also observed that the principal cardiac lesion in pregnancy was congenital in 72% of patients and in 34 women (28%) it was acquired. However, another Indian study observed rheumatic heart disease as the major contributor (84.6%) as compared with congenital heart disease (15.4%)^(21)^. The  difference can be attributed to geographic factors because congenital heart disease is found to be more common in the west, and acquired, rheumatic heart disease is more prevalent in developing countries such as India.

The present study demonstrated that the mean duration of labor was comparable in both groups. However, 52% of patients delivered during workday hours (between 8:00 am to 5:00 pm) when labor is induced, whereas only 24% of pregnant women delivered during working hours who underwent spontaneous delivery. A similar result has been shown by Oron et al.^(20)^, that 55% of women in the study group delivered during workday hours when the delivery was induced. Therefore, it is concluded that the induction of labor significantly controls the timing of delivery. The timing of delivery matters most in such cases because the team of obstetricians, anesthesiologists, and cardiologists is readily available during workday hours, which is of paramount importance in terms of maternal and perinatal outcome.

In our study, we observed that the majority (94%) had a vaginal delivery, and only one patient underwent cesarean section when labor was induced as compared with two when no induction was performed. Induction of labor does not increase the incidence of cesarean section in pregnant patients with pre-existing heart disease. Oron et al.^(20)^ observed that 63.8% had a vaginal delivery, 21.2% had cesarean section, and 14.8% had instrumental deliveries among 47 patients when labor was induced in cardiac patients, whereas 55.4% had a vaginal delivery, 33.7% had cesarean section, and 10.8% had vaginal deliveries among 74 patients with heart disease who underwent spontaneous labor. The cesarean section rate is, however, less in the present study as compared with the study by Oron et al.^(20)^ The exclusion criteria set for the current study probably contributed to the low cesarean rates. The present study has a similar rate of instrumental deliveries (18%) as observed by Oron et al.^(20)^ (15%). The comparatively higher rate of instrumental deliveries in other studies can be explained by philanthropic efforts to shorten the second stage of labor in order to avoid maternal complications. Pratibha et al.^(22)^, in their study on 200 patients with rheumatic heart disease in pregnancy, concluded that 73.5% had a vaginal delivery and 26.5% had a cesarean section. Thirty women underwent induction of labor for pregnancy-induced hypertension, pregnancy post dates, premature rupture of membranes, and term gestation. Kampman et al.^(23)^, in their study on pregnancy outcomes and deliveries in women with congenital heart disease, observed that the incidence of vaginal delivery was lower in women with congenital heart disease (76.5%) as compared with healthy women (87%), and induction of labor was more common in women with congenital heart disease (37.1%) than in controls (21.4%). Elective cesarean sections were more often performed in women with congenital heart disease (14.1% vs 1.4%), the maximum of which (36.6%) were for cardiac reasons.

Indication for cesarean sections was solely due to obstetric reasons such as fetal bradycardia, meconium-stained liquor, and deep transverse arrest in our study, and none underwent cesarean due to cardiac pathologies, which was consistent with the results of Pratibha et al.^(22)^.

There were no significant maternal complications attributable to induction of labor as well as in patients who underwent spontaneous labor in our study. Only two out of 50 patients had obstetric complications, and none of the study subjects experienced cardiac complications. In contrast, Pratibha et al.^(22)^ observed cardiac complications in 29 pregnancies (14.5%); 22 (11%) women had cardiac failure. Out of the 22 patients who had cardiac failure, 81.8% (19/22) women were in NYHA III and IV and three women (2.1%) were in NYHA class I and II developed cardiac failure later in pregnancy. However, no maternal mortality was reported in their study. The maternal complication in the form of pulmonary edema and congestive cardiac failure was seen in one case each out of 52 cases by Rangaswamy and Ramachandra^(21)^. In the present study, the relatively favorable pregnancy outcomes were attributable to the fact that the majority of the patients (96%) were NYHA class I.

In the present study, no statistically significant difference in the rate of neonatal complications between the two groups was observed. The majority of the newborns had a birth weight between 2.6-3.0 kg. There were no stillbirths, and no admissions to the NICU were witnessed in the present study. In contrast, newborn death ensued in 2.8%, and median birth weight was also comparatively lower in offspring of women with congenital heart disease in a study by Kampman et al.^(23)^. This can be attributed to the small sample size in our study as compared with their study. However, Rangaswamy et al.^(21)^ observed that only 12 out of 52 infants had birth weight <2.5 kg, seven were admitted to the NICU due to meconium aspiration (2 cases), preterm (four cases), and mild birth asphyxia (one case) and there was no perinatal mortality. Pratibha et al.^(22)^ documented 13 perinatal losses (seven IUFD, two stillbirths and four neonatal deaths), the perinatal mortality being 6.4%. The preterm and intrauterine growth retardation rates were 9.3% each. There were 76 low- birth-weight babies (37.43%). The incidence of low birth weight was 34.69% in NYHA class I and II, and 44.64% in class III and IV. Oron et al.^(20)^ found no statistically significant difference in mean birth weight between the patients who underwent induction of labor and those who had spontaneous onset of labor. A total of seven babies needed admission to the NICU. However, no stillbirth was reported.

In the index study, patients were induced with oxytocin instead of prostaglandins. Although oxytocin has to be used guardedly in patients with heart disease, we encountered no adverse effects as observed by Sau et al.^(24)^ who concluded that induction of labor with oxytocin infusion was safe and effective for patients with cardiac disease where elective delivery is warranted. Prostaglandins, however, have the disadvantage that the onset of labor is unpredictable and not clearly related to the state of the cervix. Furthermore, the tablets tend to clump together, and this may result in an uneven absorption of prostaglandin.

### Study Limitations

The limited patient numbers and the inclusion of women with only NYHA class I and II were the main limitations of the study. However, a more substantial, randomised, multicenter clinical trial is needed before definitive guidelines can be made for the induction of labor in such group of patients.

## Conclusion

This study concluded that induction of labor with oxytocin was a relatively safe procedure in women with low-risk heart disease. The induction does not result in any cardiac complications in pregnant women, and neonatal outcomes is also comparable to those who underwent spontaneous labor. More patients delivered during the daytime when electively induced, which minimized the maternal and fetal risks because the team of obstetricians, anesthesiologists, cardiologists, and perinatologists is readily available during the daytime. Thus, the results of this study may have significant repercussions for the pregnancy management of women with heart disease. Nevertheless, a more substantial, multicenter clinical trial will be indispensable before arriving at any definitive guidelines.

## Figures and Tables

**Table 1:**
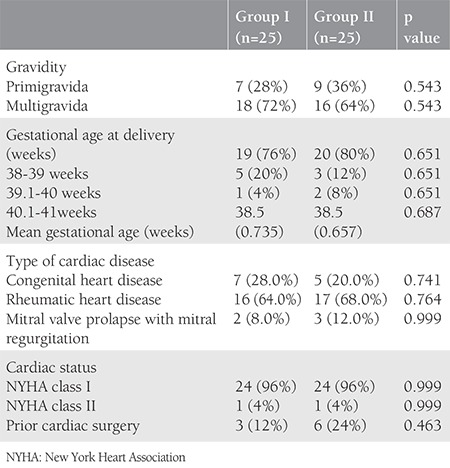
Distribution of pregnant women with cardiac disease

**Table 2:**
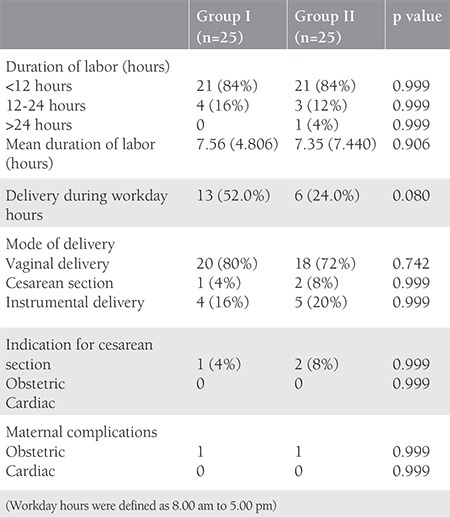
Obstetric outcomes

**Table 3:**
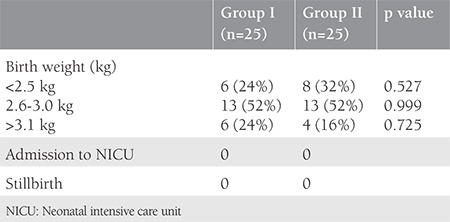
Neonatal outcome
